# Supplementary material for: Community health intervention through musical engagement (CHIME) in South Africa: A formative exploration of the feasibility and development of a music-based intervention to support perinatal mental health
Source: PLOS Glob Public Health. 2026 Feb 9;6(2):e0004878. doi: 10.1371/journal.pgph.0004878 (PMC12885302; doi:10.1371/journal.pgph.0004878)
Supplement: S1 Text — (DOCX) [file pgph.0004878.s002.docx]

**Supporting Information: Focus Group Discussion Guides**

**CHIME-SA Focus Group Discussion: Traditional Healers**

| 10:45 – 11:00 | Participants arrive |
| --- | --- |
| 11:00 – 11:20 | Consent form signing, Coffee & Tea |
| 11:20 – 11:30 | Sound check for audio recording, confirm consent for recording |
| 11:30 – 11:40 | Aims, Guides for engagement |
| 11:40 - 12:45 | Discussion  Section 1: Experience and mental health  Section 2: Group music making as an intervention  Section 3: Music in the perinatal period |
| 12:45 – 13:00 | Summary and final questions |
| 13:00 – 13:05 | End recording |
| 13:05 – 13:30 | Thanks, Vouchers and lunch |

**Aims of the session (5 minutes)**

- To find out what indigenous, participatory, community-based music-making practices already exist in SA
- To explore in what ways these existing music practices can support of emotional wellbeing or ways that it can support emotional wellbeing.
- To explore potential strategies for developing and implementing a maternal mental health intervention through indigenous music making in South Africa

**Ways to Engage in workshop (3 mins)**

- Assigning numbers for anonymity
- Encouraging everyone to join in the conversation.
- Speak one at a time and give everyone a chance to speak (hand function)
- There are no right or wrong answers
- We are curious about what you agree and disagree on
- This is an open discussion and we’re here to listen
- Any others suggested

**Discussion (+/- 70 mins)**

**Introduce PMHP and/or the importance of focusing on the perinatal period and define mental health (5 mins)**

**Section 1: Experience and mental wellbeing (35 mins)**

1. Could you introduce yourself and define your role as amagqihra/healers?
2. Can you tell us about how your healing process works for people experiencing mental health problems?

Provide examples of people experiencing common mental health problems: constant worries, lack of sleep, feeling tired, feeling low and sad etc.

1. Have any of you had any experiences of using music in your healing/practice? If so can you tell us about it?

Probes: If people don’t use music in their practice, why not? How helpful is music in their healing practice?

Probe for music type, instrumentation, improvisation, lyrics, participation of client; how music is introduced into the practice

**Section 2: Group music making as an intervention (15 min)**

1. Can you tell us about the relationship between music and healing?
   1. How is music shared in your culture?
   2. How is the music shared in cultural ceremonies different from the music shared in your sessions?

Probe about the benefits of music and culture, social connectedness, mental wellbeing and resilience building

1. Can music help people deal with their stress, worries, and concerns? If so, how? Can you give any examples?
2. What do you think about singing or making music as a way to help with **mental health problems**? Can you give examples of music practices you know about, that specifically help with emotional or mental well-being?

Probe only if necessary: What kind of shared singing, or shared healing practices exist? Are there other music practices you think would be helpful for mental wellbeing?

**Section 3: Music in the perinatal period (35 mins)**

1. Do you know of any traditional ceremonies that are performed for pregnant women or new mothers? Please tell us about them.
   1. Are there any songs are dances performed at these ceremonies?
   2. Are fathers or other family members included in these ceremonies?
   3. What is the purpose of these ceremonies, how do they help the mother?
2. What kinds of traditional ceremonies are performed for babies?
   1. What is the purpose of these ceremonies?
   2. Are there any songs performed at these ceremonies?
   3. Do these ceremonies bring people together, if yes, can you give a few examples?

Probe if necessary: How do these ceremonies/songs improve the social connections, resilience, support for mothers/babies?

**CHIME-SA Focus Group Discussion: Ethnomusicologists**

| 13:20 -13:30 | Meeting link opens, test to see if recording is working SS |
| --- | --- |
| 14:00 – 14:10 | Participants join |
| 14:10 – 14:30 | Informed consent and signing forms SS |
| 14:30 – 14:31 | Check for recording and gallery view SS |
| 14:31 – 14:40 | Introductions (SH); Aims (SS), Rules of engagement (SS) |
| 14:40 – 14:45 | Ice breaker (SS) |
| 14:45 – 15:45 | Discussion () |
| 15:45 – 15:55 | Summary, anything else? |
| 15:55 – 16:00 | Goodbye |
| 16:05 | SS to stop recording |

**Aims of the session PPT (5 minutes)**

- To explore the landscape with respect to existing participatory community music-making practices in SA
- To explore whether any of these are supportive of emotional wellbeing or potentially could be adapted to support emotional wellbeing.
- To explore potential strategies for developing and implementing a maternal mental health intervention through indigenous music making in South Africa
- To explore whether a participatory music intervention for perinatal mental health, delivered by CHWs, is feasible in South Africa with respect to several domains of feasibility: acceptability, demand, likelihood of implementation, adaptability, ability to be integrated into routine procedures, possibility for expansion
- To generate suggestions for methods and processes to be used in a co-design workshop (Part B) for developing a prototype repertoire of songs.

**Rules of Engagement**

- Encouraging active participation
- Speak one at a time and give everyone a chance to speak (hand function)
- There are no right or wrong answers
- We are curious about what you agree and disagree on
- This is an open discussion and we’re here to listen
- Any others suggested

*************ICEBREAKER***************

What is your favourite childhood song? Why?

**Discussion (75 mins)**

**Introduce the section: Music and experience**

1. What is your area of expertise?
2. What kind of music do you play or sing?
   1. What are the main elements of your performance style?
3. Do you sing or play music in a group?
   1. What kinds of group based music activities do you engage in?

**Introduce the section: Group music making as an intervention**

1. Can music help people deal with their stress, worries, and concerns? If so, how?
   1. Can you give any general examples from your own experience?
2. What do you think about singing in group as a type of music making activity for mental health problems?
   1. What kind of group singing, or group healing practices exist? Choirs? Other groups in the community?
   2. Are there other music practices you think would be helpful for mental wellbeing?

**Introduction to the section: Music in the perinatal period**

1. What kinds of songs are sung to babies? Follow up: Can you sing any of these songs?
   1. Who knows these songs and who sings them to babies?
   2. What is the benefit of these songs/ why are these songs performed?
2. Do you know of any songs and dances that are performed at ceremonies for pregnant women or new mothers? Please tell us about them.
3. Do you think singing together would be beneficial for women who are experiencing stress, anxiety, or depression during and after pregnancy? If so, why? If not, why not?
4. What kind of singing or music do you think would be beneficial (if any)?
   1. Would it be beneficial to teach pregnant mothers and new mothers lullabies to sing to their babies?
5. What group-based musical activities do you think might help women deal with stress, anxiety, and depression if they came together regularly during pregnancy/after delivery?

**Introduction the section: Proposing community music making for perinatal mental health**

1. Do you think choirs or singing groups that are specifically for pregnant women and or new mothers would be helpful?
   1. What if they sang songs with words that were about what you can do if you are feeling anxious or depressed? Would this be helpful? Why or why not?
   2. Who should lead these groups?
   3. Do you think it is a good idea to include lullabies? Why or why not?
   4. Do you think chorus singing (using voice) would be a helpful type of music making activity or is there another music practice you think would be better?
   5. Do you think it would be helpful to include father/partners or other family members at times or not?
   6. What else do you think we could include in a program like this to make sure it is helpful?

**Introduction to the section: Introducing CHIME and Mankosi workshop (20 mins)**

- PPT for CHIME and Workshop (5 mins)

1. We would value your advice on improving our co-development Workshop in Mankosi.
   1. What can be improved in the workshop aims?
      1. Are these aims achievable?
      2. What do you think we are missing?
   2. What can be improved in the workshop method?
      1. Do you think the processes we are planning for the workshop method encourages participants to design the intervention? How could our methods be improved?
      2. Are there other approaches you could suggest that we use to encourage co-development between participant and researcher?
      3. What should we be aware of/ cautious of?
   3. What can be improved in the workshop structure?
      1. How do you think the co-development design can be improved?
      2. Do you think the structure aligns with the aims of the workshop?

Summary - 3 mins

Anything missing, anything that we may have misunderstood, any ideas or changes of mind - 3 mins

**CHIME-SA Focus Group Discussion: Health workers Group**

| 09:10 – 09:20 | Meeting link opens, test to see if recording is working |
| --- | --- |
| 09:30 – 09:40 | Participants join, check for recording and gallery view |
| 09:40 – 09:50 | Introductions, Aims and Guide for engagement |
| 09:10 – 10:50 | Discussion |
| 10:50 – 11:00 | Debrief and closing remarks |

**Introductions, description of CHIME (5 minutes)**

**Aims of the session PPT (3 minutes)**

- To explore the landscape with respect to existing indigenous, participatory, community-based music-making practices in SA.
- To explore in what ways these are supportive of emotional wellbeing or potentially could be adapted to support emotional wellbeing.
- To explore challenges and opportunities, positive examples or strategies developed with respect to health workers delivering a community based music intervention
- To explore the landscape with respect to existing participatory community music-making practices in primary healthcare facilities
- To establish whether any of these are supportive of mental health or potentially could be adapted to support mental health.
- To explore potential challenges and strategies for implementing a maternal mental health intervention through traditional music making led by CHWs or primary healthcare workers.
- To establish whether a participatory music intervention for perinatal mental health, delivered by health workers, is feasible in South Africa with respect to several domains of feasibility: acceptability, demand, likelihood of implementation, adaptability, ability to be integrated into routine procedures, possibility for expansion

**Ways to Engage in workshop (2 mins)**

- Encouraging everyone to join in the conversation.
- There are no right or wrong answers
- We are curious about what you agree and disagree on
- This is an open discussion and we’re here to listen
- Please put your phones on silent
- Any others suggested

**Discussion (60 mins)**

**Ice breaker: (5 mins)**

Could you please tell us a bit about the role that music plays in your life currently?

**Section 1: Challenges to mental wellbeing (10 minutes)**

1. What kinds of mental health or psychosocial support do you know of that women access during and after pregnancy? (Probe for location, providers, accessibility)
2. What group-based activities do you think might help women deal with stress, anxiety, and depression if they came together regularly in the perinatal period? (Probe for facility based and community-based activities, formal and informal, cultural)

**Section 2: Music as an intervention for mental health (10 mins)**

1. Can music help people deal with their stress, worries, and concerns? If so, how?
2. Do you think singing together would be beneficial for women who are experiencing stress, anxiety, or depression during and after pregnancy? If so, why? If not, why not? Probe for how the group singing may work.

**Section 3: Existing music practices in healthcare (15 mins)**

1. Do you know of any existing music practices in facility-based healthcare settings or community based settings? Can you describe them?
   1. What are some of the effects of these practices?
   2. Can these practices be adapted to support mental wellbeing?
2. If there is a lack or scarcity of these practices, can you comment on the reasons for this: what are barriers to including musical practices for health.
3. Would it be beneficial to include perinatal women in new or existing music practices in a clinic-based setting? How would you see this working? (Probe for logistics, facilitators, barriers and enablers)

**Section 4: Implementing a community music making intervention using CHWs (15 mins)**

**Describe outcomes of FGDs done to date and the Mankosi co-design workshop.**

1. What are your thoughts on this potential intervention?
   1. Do you think it is possible for a music intervention such as this (or similar) to be implemented in the maternity facilities or MCH facilities ? Why or why not?
   2. Who should lead these groups? (Probe for advantages and disadvantages of using facility staff versus CHWs)
   3. Do you think it would be helpful to include women’s partners or chaperones in this intervention?
   4. What would be required to support the success of such a programme?

**CHIME-SA Focus Group Discussion: One-to-One Mentor Mothers**

| 10:00 – 10:10 | Sound check for audio recording, confirm consent for recording |
| --- | --- |
| 10:10 – 10:20 | Aims, Guides for engagement |
| 10:20 – 11:20 | Discussion  Section 1  Section 2  Section 3  Section 4 |
| 11:20 – 11:30 | Summary and final questions/comments |
| 11:30 – 11:35 | End recording and save to drive |

**Aims of the session PPT (5 minutes)**

- To explore challenges and opportunities, positive examples or strategies developed with respect to community health workers delivering services to women through One to One
- To establish whether any of these are supportive of mental health or potentially could be adapted to support mental health.
- To explore the landscape with respect to existing indigenous, participatory, community-based music-making practices in SA
- To explore in what ways these are supportive of emotional wellbeing or potentially could be adapted to support emotional wellbeing.
- To explore potential strategies for developing and implementing a maternal mental health intervention through indigenous music making in South Africa
- To explore whether a participatory music intervention for perinatal mental health, delivered by CHWs, is feasible in regions across South Africa with respect to several domains of feasibility: acceptability, demand, likelihood of implementation, adaptability, ability to be integrated into routine procedures, possibility for expansion

**Guide for Engagement**

- Encouraging active participation
- Speak one at a time and give everyone a chance to speak (hand function)
- There are no right or wrong answers
- We are curious about what you agree and disagree on
- This is an open discussion and we’re here to listen
- Any others suggested

**Discussion (75 mins)**

**Section 1: Perinatal mental health in Mankosi (Barriers and challenges)**

1. What kinds of emotional challenges do women encounter during pregnancy and after delivery?
   1. Follow up questions – what causes this challenge? What are the consequences? why is it a problem?
2. Do women sometimes experience mental health problems (e.g. sadness, worry, fear) during pregnancy or after they have given birth?
3. What are the possible effects of mental health issues during and after pregnancy? How severe can it be? (for the mother and her baby, others in her family or community)

**Section 2: Existing strategies for coping**

1. What kinds of things do women typically do to help themselves if they experience anxiety, stress, or depression?
2. Is there any way to help prevent women experiencing mental health problems during and after pregnancy?
3. Is there any treatment for mental health conditions during and after pregnancy? If so, how effective is this treatment thought to be?

**Section 3:**

1. What kinds of emotional support do health workers offer to women during and after pregnancy?
   1. Follow up on each point.
2. What group-based activities do you think might help women deal with stress, anxiety, and depression if they came together regularly during pregnancy/after birth?

**Section 4: Proposing community music making for perinatal mental health**

1. Do you think singing together would be beneficial for women who are experiencing stress, anxiety, or depression during and after pregnancy? If so, why? If not, why not?
2. What kind of singing or music do you think would be beneficial (if any)?
   1. Would it be beneficial to teach pregnant mothers and new mothers’ lullabies to sing to their babies?
3. Do you know of any kinds of songs and dances that are performed at ceremonies for pregnant women or new mothers? Please tell us about these.
   1. Who performs the music?
   2. Who is the music intended for?
   3. What is the benefit of these songs/ why are these songs performed?
4. Do you think choirs or singing groups that are specifically for pregnant women and or new mothers would be helpful?
   1. Who should lead these groups?
   2. What if they sang songs with words that were about what you can do if you are feeling anxious or depressed? Would this be helpful? Why or why not?
   3. Do you think it might be beneficial for some women and not others?
   4. Do you think choirs or singing groups are the best type of music making activity or is there another music practice you think would be better?
   5. Do you think it would be helpful to include father/partners or other family members at times or not?

**In closing**

Anything missing, anything that we may have misunderstood, any ideas or changes of

**CHIME-SA Focus Group Discussion: One to One Management**

| 09:10 – 09:20 | Meeting link opens, test to see if recording is working SS |
| --- | --- |
| 09:30 – 09:40 | Participants join |
| 09:40 – 10:00 | Informed consent and signing forms SS |
| 10:00 – 10:01 | Check for recording and gallery view SS |
| 10:01 – 10:10 | Introductions (SH); Aims (SS), Rules of engagement (SS) |
| 10.10-10.15 | Ice breaker (SS) |
| 10:15 – 11:30 | Discussion (SH to lead, SS to co-f)  Challenges to mental wellbeing 10.15-10.25  Solutions to mental health challenges 10.25-10.35  Music as an intervention 10.35-10.45  Music in the perinatal period 10.45-11.00  Proposing Community music making 10.00-10.10  Introducing CHIME and workshop 10.10-10.30 |
| 11:30 – 11:40 | Summary, anything else? |
| 11:40 – 11:45 | Goodbye |
| 11:45 – 11:50 | Emma to send recording |

**Aims of the session PPT (5 minutes)**

- To explore challenges and opportunities, positive examples or strategies developed with respect to community health workers delivering services to women through One to One
- To explore the landscape with respect to existing participatory community music-making practices in area served by One to One
- To establish whether any of these are supportive of mental health or potentially could be adapted to support mental health.
- To explore potential challenges and strategies for implementing a maternal mental health intervention through traditional music making led by CHWs at One to One
- To establish whether a participatory music intervention for perinatal mental health, delivered by CHWs, is feasible at One to One with respect to several domains of feasibility: acceptability, demand, likelihood of implementation, adaptability, ability to be integrated into routine procedures, possibility for expansion
- To generate suggestions for methods and process to be used in a co-design workshop for developing a prototype repertoire of songs.

**Rules of Engagement**

- Encouraging active participation
- Speak one at a time and give everyone a chance to speak (hand function)
- There are no right or wrong answers
- We are curious about what you agree and disagree on
- This is an open discussion and what you say will not affect your employment
- Any others suggested

*************ICEBREAKER***************

What is one of your favourite songs? Why?

**Discussion (75 mins)**

**Introduce the section: Challenges to mental wellbeing (10 minutes)**

1. What kinds of emotional/psychological/mental health/psychosocial challenges do you think women experience during pregnancy and after delivery in SA and also in Mankosi, in particular? Probe for presentations, causes and outcomes

**Introduce the section: solutions to mental health challenges (10 mins)**

1. What kinds of mental health support can women access during and after pregnancy in Mankosi and surrounds ?
2. Is there any treatment for mental health issues during and after pregnancy in Mankosi and surrounds? If so, how effective is this treatment thought to be?
3. What kinds of things do women in these areas usually do to help themselves if they experience anxiety, stress, or depression?
4. What group-based activities do you think might help women deal with stress, anxiety, and depression if they came together regularly during pregnancy/after delivery?

**Introduce the section: Music as an intervention (10 mins)**

1. Can music help people deal with their stress, worries, and concerns? If so, how?
2. What do you think about singing in group as a type of music making activity for mental health problems?
   1. Choirs? Other groups in the community?
   2. Are there other music practices you think would be helpful?

**Introduction to the section: Music in the perinatal period (15 mins)**

1. What kind of singing or music or music making do you think would be helpful for pregnant women or new mothers (if any)?
2. Do you think singing together would be beneficial for women who are experiencing stress, anxiety, or depression during and after pregnancy? If so, why? If not, why not?
   1. Do you think it might be helpful for some women and not others?
   2. Do you know of any kinds of songs, other music or dances that are performed at ceremonies for pregnant women or new mothers? Please tell us about them.
3. Do you know of any kinds of songs that are sung to babies?
   1. Who knows these songs and who sings them to babies?
   2. What is the benefit of these songs/ why are these songs performed?
   3. Would it be helpful to teach pregnant mothers and new mothers’ lullabies to sing to their babies? In what way would it be helpful?

**Introduction the section: Proposing community music making (10 mins)**

1. If we were to create singing groups specifically for pregnant women and or new moms - how best could we design the music content and the way the music happens?
   1. What if they sang songs with lyrics that were about what you can do if you are feeling anxious or depressed? Would this be helpful? Why or why not?
   2. How do you think these groups could work through the One to One model? How could these groups function to involve women in the community who may find them helpful?
   3. What would be the challenges? What would be the opportunities?
   4. Do you think it would be helpful to include father/partners or other family members in the music making at times or not?
   5. What else do you think could be included in a community music making to make sure it is helpful?

**Introduction to the section: Introducing CHIME and Mankosi workshop (20 mins)**

- PPT for CHIME and Workshop (5 mins)

1. We would value your advice on improving our co-development Workshop in Mankosi.
   1. What can be improved in the workshop aims?
      1. Are these aims achievable?
      2. What do you think we are missing?
   2. What can be improved in the workshop method?
      1. Do you think the processes we are planning for the workshop method encourages participants to design the intervention? How could our methods be improved?
      2. Are there other approaches you could suggest that we use to encourage co-development between participant and researcher?
      3. What should we be aware of/ cautious of?
   3. What can be improved in the workshop structure?
      1. How do you think the co-development design can be improved?
      2. Do you think the structure aligns with the aims of the workshop?

Summary - 3 mins

Anything missing, anything that we may have misunderstood, any ideas or changes of mind - 3 mins
