# Supplementary material for: Community health intervention through musical engagement (CHIME) in South Africa: A formative exploration of the feasibility and development of a music-based intervention to support perinatal mental health
Source: PLOS Glob Public Health. 2026 Feb 9;6(2):e0004878. doi: 10.1371/journal.pgph.0004878 (PMC12885302; doi:10.1371/journal.pgph.0004878)
Supplement: S2 Text — (DOCX) [file pgph.0004878.s003.docx]

**Supplementary Information: Positionality Statement**

Our team is comprised of researchers and academics from South Africa, the United Kingdom, Australia and the United States. We are cognisant of the privilege and under representation of indigenous South African identities within our team considering the cross-cultural research we are conducting. The two researchers who co-facilitated the Mankosi Eastern Cape workshop are fluent in their indigenous home languages.

Ncumisa Waluwalu (NW) is an established research assistant with a research organisation in the Eastern Cape and has facilitated in multiple projects for academics and health organisations conducting research in the area. She lives very close to Mankosi, and is a first language isiXhosa-speaker and is familiar with our targeted participants. She has a working relationship with members of the community in Mankosi and with the NGO we recruited from for this work.

Siphumelele Sigwebela (SS) is a junior researcher whose home language is isiZulu. However, she understands isiXhosa well and can speak the language with moderate fluency. SS has experience in academic and lab-based research.

The researchers who are not South African have demonstrated success in deeply respectful approaches in their cross-cultural work in other settings (e.g., indigenous Australians, The Gambia). In the nine months of preparation of this research, they worked with the South African team in a collaborative and highly supportive manner without imposing any pre-conceived agenda. They have generated funds to contribute to the costs of the research including the salary of SS and all the costs pertaining to the workshop, transcriptions and translations.

Below is a table outlining the qualifications, background and known biases of each member of our research team.
