# Supplementary material for: Community health intervention through musical engagement (CHIME) in South Africa: A formative exploration of the feasibility and development of a music-based intervention to support perinatal mental health
Source: PLOS Glob Public Health. 2026 Feb 9;6(2):e0004878. doi: 10.1371/journal.pgph.0004878 (PMC12885302; doi:10.1371/journal.pgph.0004878)
Supplement: S1 Table — (DOCX) [file pgph.0004878.s004.docx]

**Supporting Information: Positionality Table**

| Name | Qualification | Background | Bias |
| --- | --- | --- | --- |
| Siphumelele Sigwebela | BA Psychology (Honours)  BA English  BA Chinese Studies | Masters candidate, IsiZulu home language. I have trained in mostly Cognitive psychology dealing with emotion elicitation, cross-cultural research and maternal mental health. I have skills in Bio physiology, technology and digital media platforms. I have co-supervised on computer science honours projects and designed virtual environments. | I am not an isiXhosa woman, while many Nguni traditions are similar, I know that my knowledge of isiXhosa traditions is limited. Although my family is traditional, I have a limited experience of rural life, having only lived in rural KZN for 4 weeks a year since childhood. I have not experienced motherhood or pregnancy. My perspective is that of a young woman who has no dependants. I know that my privilege and education may result in my perspective being completely different from the participants. I am an investigator while I may be curious, I am aware that the questions and discussions pertain to participants real lives and experience. I may have a belief or perspective that is not appreciated by the participants. |
| Ncumisa Waluwalu | Matric Certificate  Research Methods certification. | Administrative assistant trained in Qualitative research methods. Qualitative interview techniques, preventing TB transmission, Nutrition, Child and Maternal Health | I have worked with this organisation before, I was last there in September. I think we got on very well and the ladies understood me. I don’t think I’ll have any problems when we are there for the workshop. |
| Simone Honikman | MBChB, Dip Obs, DCH, DFFP, MPhil (MCH) | Associate Professor, founder and director of Perinatal Mental Health Project (PMHP), UCT (project launched 2002). Medical doctor, researcher, trainer, teacher. Involved in research, training, policy and guideline development, knowledge translation, advocacy and service design. Experience working with researchers and frontline providers in Nepal, India, Ethiopia, Malawi, Uganda, Lesotho, Botswana. | I am a white South African woman who was greatly privileged by the South African Apartheid regime and am acutely aware of the continued benefit and privileges I have access to through this distorted legacy. I have dedicated my career to working for women who have been marginalised by intersecting systems of oppression, including patriarchy, an often-abusive health system, racism and poverty.  My knowledge of isiXhosa is rudimentary. I have worked closely with isiXhosa-speaking health providers, both in urban and rural settings around South Africa for my entire career. My relationships with the providers have taken many forms, depending on the work setting, but have almost always enjoyed a warm, respectful and collaborative engagement.  I have worked closely with One to One Africa for nearly two years. The Nyamekela 4 Care (N4C) intervention that was developed by the PMHP was introduced to One to One early in 2020. It was presented as an offering to embed into routine team meetings of Community Health Workers (CHWs), peer-driven support, knowledge and skills development and self-care. Over many months, I worked closely with One to One management to adapt the intervention to best fit the organisation’s needs. This culminated in a face-to-face training workshop, which I co-facilitated with One to One staff in June 2021. Thereafter, I have been providing distance-based mentoring support for One to One staff to run their own N4C meetings. Preliminary evaluation of the intervention shows extremely high levels of support and uptake of N4C with staff taking initiative to adapt the intervention as they see fit and to discuss openly at the mentoring sessions, the challenges they experience. It was through this work that I came across the existing practice of One to One CHWs to sing songs about common health problems in their communities. These songs appear to fulfil several functions: a practice for entertainment and joy, a practice to remind each other of health information, a practice to transmit health information to clients.  When I enquired from CHWs, their supervisors and managers, whether songs could be developed around the topic of maternal mental health, there was enormous interest in the idea. The notion of PMHP researchers trying to understand more about their music-making processes and co-developing songs together appeared to be perceived as an affirmation of their skills and practice – a recognition of the value of interventions developed by local providers. Discussion of the potential for these songs to be used by other health providers across South Africa was also seen to be an exciting and valuable proposition both by frontline workers and management staff. |
| Sally Field | MA, Psychology (Hons) | Project Co-ordinator Perinatal Mental Health Project. Involved in research, guideline development, knowledge translation, advocacy, service design, monitoring and evaluation. Experience in qualitative research methods. | I am a white South African woman from an advantaged background. I have training in community development and participatory practices and try to be sensitive to cultural and power dynamics. My role in this research project is largely administrative and advisory. I will not be an active participant in the field. |
| Vivette Glover | MA, PhD, DSc | Visiting Professor of Perinatal Psychobiology, Imperial College London  Expert in perinatal mental health, the effects on the chid, and the underlying biological mechanisms.  Published over 300 papers in peer reviewed journals. H-index 73.  Advisor to UK government on these issues. | I am a white older English woman. I have been involved in research in different parts of the world including The Gambia, and I am very aware of cultural differences in understanding mental illness,  My contribution to this research will be on the overall design of the project, and the methods used for analysis. |
| Bonnie McConnell | PhD, Ethnomusicology Graduate Certificate, Global Public Health | Senior Lecturer in ethnomusicology, Australian National University Expertise: Ethnography of music; music and health research; participatory methodologies; gender studies. My research focuses on the integration of indigenous knowledge and communication systems in global health programs. I have been conducted collaborative ethnographic research with performers and health workers in the Gambia and Tanzania since 2006. | My background and position as a white Australian/American woman who has spent 7 years in eastern and western Africa (Tanzania and The Gambia) shapes my perspective, gives me a position of privilege, and presents potential biases. My role on the project is to provide disciplinary expertise and guidance on the collaborative co-design process, drawing on the approach that we developed in the Gambia. Based on my prior experiences, I am aware of the potential challenges of cross-cultural research teams, and the need to be careful not to assume that experiences from elsewhere will be mirrored in the South African context.  It will be essential for me (and the entire team) to be sensitive to power differentials at each stage of the project, to ensure that indigenous perspectives are valued, and indeed leading the research process, to develop outcomes that are aligned with the experiences and needs of the target community. |
| Lauren Stewart | PhD Cognitive Neuroscience of Music | Professor in Psychology, Goldsmiths University London  Music cognition and cognitive neuroscience of music; application of this knowledge to areas of physical and mental health including the design of the CHIME mental health intervention for the perinatal period. I was the principal investigator on a funded project based in the Gambia, to which the current work relates. | I am a white British woman, based in the UK. I have never visited South Africa and my knowledge of the cultural context in which the work will take place has been gleaned through conversations with the other team members, particularly SS and SH. I was involved in designing qualitative investigations with pregnant women and local women’s groups in The Gambia and I have experience of leading work with a highly international and interdisciplinary team where we consulted widely with community groups to ensure that our work took account of local beliefs, frameworks and customs. I am aware that the context in which we are working in South Africa will provide different affordances, contexts and frameworks. I am also aware of the need to be aware of power differentials that can exist in this work and to be sensitive to this at each stage of the project. |
| Katie Rose M. Sanfillipo | BA Psychology BA Music MSc Music, Mind and Brain PhD Psychology | Research fellow, Centre for Healthcare Innovation Research, City St George’s, University of London.  Expertise in qualitative and quantitative research methods, intervention development, maternal mental health, and healthcare innovation implementation.  I am currently part of a research group at City working to promote the decolonisation of health research.  I completed my PhD in 2020 where I worked collaboratively with local women’s groups and local partners to co-develop a community based music intervention (CHIME) to support maternal mental health in The Gambia. | I am aware of my positionality as a white, American, young adult female that does not have children. I am aware of the importance of recognising and reflecting upon this throughout my research activities. I am very much an outsider; I have never been to South Africa and have very rudimentary understanding of its culture.  My experience has been working within The Gambia where I spent time living and working. Even there I made sure to be reflexive throughout the research process and relied on the local RAs, who are native to The Gambia, to guide me and champion the research. Aware of the power-dynamics when I am present, I attended very few research activities in The Gambia (FGDs and intervention sessions).  For this project, I see my role as one of an advisor based on the experience and work I have done within The Gambia. I do not plan to be present for any of the FGDs or workshops. I see this project as a form of knowledge exchange, where we will be working collaboratively with the experts in this area in South Africa (One-to-One and the South African research team members), learning from each other’s different perspectives and expertise, while ensuring the aims of the research are rooted in the needs the community this project hopes to serve. |
