# Supplementary material for: Community health intervention through musical engagement (CHIME) in South Africa: A formative exploration of the feasibility and development of a music-based intervention to support perinatal mental health
Source: PLOS Glob Public Health. 2026 Feb 9;6(2):e0004878. doi: 10.1371/journal.pgph.0004878 (PMC12885302; doi:10.1371/journal.pgph.0004878)
Supplement: S2 Table — (DOCX) [file pgph.0004878.s005.docx]

| Themes | Quotes |
| --- | --- |
| **Theme 1: Challenges that contribute to mental distress** | “So I think emotionally it’s the things that we kind of expect in the relationships then later on they turn out not to be what we really, really expected and then once it doesn’t work out of course one will go to the tavern and drink a lot of alcohol, take drugs yeah and end up leaving home. Because whatever that you were expecting didn’t go the way that you were expecting it to go.” – One to One management |
| 1.1 Social determinants of poor mental health | so if you look at statistics of age range of people who are having babies right now it’s no longer the traditional women or let me put it this way: women who attend mostly free clinics and governments er…spaces are not necessarily women who are business or professionals it’s poor people. It’s everyday people who…who rely on government grants and SASSA funding and stuff like that which means more than often, they are…they don’t have access to information, they don’t have access to…to that…that high level health care system right so they rely on whatever the government has to offer. -Musician |
| 1.2 Unhelpful coping strategies | “It’s just that now our communities are no longer set up as they were traditionally in…in our homesteads like where I am in Newcastle it’s transformed completely from what it was before now more of township set up where you have more shebeens that schools more prisons than there are community centres so people have lost their sense community and therefore putting a lot of pressure on their mental health and how they gonna make it through and…and now they have these social worker schemes where they provide people with food whereby there is so much land that’s available people can grow their own food but somehow they have been robbed of that power to sustain themselves but now they need to be dependent on somebody else to make sure that they are sustained.” -Musician  “I know it’s happening generally everywhere, you find out that some, they get pregnant while they were not ready to be pregnant and now the partner is not there to give the kind of support. The woman herself is not in a state where she can be able to support herself. The woman will spend time thinking about, how am I gonna afford this child. So, now that…that leads to lots of stress now which in most cases you find out they end up… drinking while pregnant or either using drugs while pregnant because they are trying to avoid the stress and not having the alternative way”– One to One management |
| 1.3 Stigma and isolation | “When for example, a person has stress…the person would say I drank or went to the shebeen and then it gets bearable. It doesn’t get bearable, instead… you are adding. Because, there you arrive and drink maybe there are guys at the shebeen … I mean she is not dating this guy. She will be won over by this guy and go and sleep with him, and the next day, alcohol is gone; that thing comes back because as she slept with that guy, the people who saw her that time being won over …and the whole village knows…So, it is adding stress on top of the one she already had” -Mentor Mother |
| **Theme 2: Community music practices in South Africa** | “Well, firstly, maybe I shouldn’t say that because everyone is musical and then we all musicians in many ways.” – Musicologist  “Africans don’t talk about music they just make music, as we are speaking now, they are singing there.” - Musicologist |
| - 1. Music is embedded to South African culture | “And you will see that this person she is singing as song and is referring to something that has just happened. And to someone that has also er.. made her to feel this way. So, in that way you will hear that she is trying to kind of passing a message and even if that person is around. You will hear that ok this person is referring to what happened earlier on between the 2 of us. And yes… so I have seen music in my community the way that most women use as a tool of kind of responding or sharing or impressing how they felt at a particular moment.” – One to One management  “Because singing is so much much a part of er… the culture, and, ther is, I think there is a more homogenous group of.. of clients. Certainly in Makhanda in the clinics, who generally speak the same language. Er… although there maybe, you know religious differences there. I think there is.. there is a shared.. there is a lot shared there” – Health worker |
| 2.2 Cultural change | “Now, when ugogo have all these lullabies in her mind she passes away, all that is gone. You understand? So we need to keep records!” – Traditional healer  “We are in the industrial days where women go to school, they don’t go to the fields, so they don’t know how to sing those songs… So there is no time for those songs now, there is only time for TVs and loud music- that is not therapeutic for them, that has got after effect for them.” -Traditional healer  “Also to keep the very same music in records, in written records so that we keep it for the future because we…we somehow lose these things because like right now we are going to have to resuscitate these old songs” – Traditional healer |
| 2.3 Musical practices associated with perinatal health | “We keep the people like senior female elder- the midwives who knows those songs- those are pre-labour songs we talkers about that you have to sing those songs perform this process of giving labour to this child.” -Traditional healer |
| **Theme 3: Benefits of music making** | “As for me, I think one of the thing that could help is for them to for a support group, those who are pregnant and the ones who have delivered. Where there will be fun [*Apho kengoku kuzakonwatywa khona*]”– Community health workers |
| - 1. Building social connection | “And you will see that this person she is singing as song and is referring to something that has just happened. And to someone that has also er.. made her to feel this way. So, in that way you will hear that she is trying to kind of passing a message and even if that person is around. You will hear that ok this person is referring to what happened earlier on between the 2 of us. And yes… so I have seen music in my community the way that most women use as a tool of kind of responding or sharing or impressing how they felt at a particular moment.” – One to One management  where you discover, my problem is smaller compared to…I thought mine was bigger. It is smaller because [*niphefumelana*], the other finds out that… it is bigger than mine. In so doing, each and every person feels free now and see that, ‘No maarn, let me join the other kids, [andilohlazo maarn] I am the same as the other kids’. ” – Traditional healer |
| - 1. Transformation | “I don’t know what context you are thinking of running these singing groups but I know that singing in a hospital setting is really powerful…thing to do kind of to take over that space with your voices and make it your space again.”- Musicologist  “The act of singing together create a sense of community and it give a chance for people to create a community with each other without… in a space where they wouldn’t have felt like they are in community with each other” – Musicologist  “So that is where I saw the power of music and came to the conclusion that music does not heal but prepares the mind for the healing ” – Musicologist  “And I have also seen, music is also such a great motivator even if there is an event where there is no planned music, every time people come up to speak, one will just come up with a song and everyone follows so now you get that kind of motivation for a person to be able to speak up after the song. So, I know it for me as well, it’s such a motivator if I have to say something then there is a song.. either I start a song that can give myself that kind of power or strength to talk so people also use it as that of.. what can I say, motivator or yeah… to speak up” – One to One management |
| **Theme 4: Making music to support mental health** |  |
| 4.1 Ingredients of music | “obviously on Radio and TV we have all this gqom, house and hip-hop but there is an element of self-identity that is missing”- Musician  “The more fundamental way of using music which uses the material of what musicing is that itself; it has the potential to bring people together to have an experience, because it’s a shared experience… you don’t have to have very direct messages… for people to overcome particular problems…” – Music Educator |
| 4.2 Contexts of music making | “My initial feeling was the clinic is not ideal, in terms of how busy it is … even just getting to the clinic sometimes, depending on the location of the clinic, can be quite challenging. [But] …I know in our context …we found that as long as you partner with the clinic, good things can come from that. Trying to set something up completely separate, it often just doesn’t get off the ground …as a route in, it could be great. And to try and keep that connection, in terms of space and busyness it might have to happen in a different location. But, not to lose that link, I think, is quite important.” – Health Worker  “Singing in a hospital setting is really powerful… to take over that space with your voices and make it your space again…the act of singing together creates a sense of community …in a space where they wouldn’t have felt like they are in a community with each other.” – Health Worker |
