# Supplementary material for: Community health intervention through musical engagement (CHIME) in South Africa: A formative exploration of the feasibility and development of a music-based intervention to support perinatal mental health
Source: PLOS Glob Public Health. 2026 Feb 9;6(2):e0004878. doi: 10.1371/journal.pgph.0004878 (PMC12885302; doi:10.1371/journal.pgph.0004878)
Supplement: Attachment — Submitted filename: REVIEW REPORT pgph.pdf [file pgph.0004878.s006.pdf]

REVIEW REPORT

PLOS Global Public Health
Community Health Intervention through Musical Engagement (CHIME) in South Africa: A formative investigation of the feasibility and development of a music-based intervention to support perinatal mental health by Siphumelele Sigwebela, Bonnie B McConnell, Ncumisa Waluwalu, Thandi Davies, Katie Rose M. Sanfilippo, Lauren Stewart, Sally Field, Vivette Glover, Simone Honikman

Manuscript number: PGPH-D-25-01381

The study is both timely and essential in the context of perinatal care, particularly when there is a growing need to refocus efforts toward improving the quality of perinatal mental health services, especially in sub-Saharan Africa. It introduces accessible and cost-effective interventions that are already embedded within community settings. If appropriately adopted and tailored to perinatal mental health, these interventions have the potential to significantly improve maternal health outcomes.

Section by Section Comments:

1. **Title**
   The title is generally informative. However, given the qualitative nature of the findings, the study appears to be more exploratory than investigative. I recommend rephrasing the title to reflect this, for example: *"...a formative exploration of the…"* Additionally, the CHIME intervention should be clearly defined or operationalized, as it is currently not well explained.

2. **Abstract**
   The abstract is clear, concise, and well-written.

3. **Introduction**
   The introduction is informative and aligns well with the topic of the study. However, please note:
   - Line 58: There is a typographical error in *"the Gambia"*.
   - Line 95: Future tense is used and should be revised for consistency.

4. **Methods**
   The methodology is well detailed, providing enough information to ensure reproducibility.
   The study population is well described under "Study Site and Sample." However:

- The description of the study site is minimal. I suggest the authors provide a more comprehensive overview of the study site, including relevant characteristics, to justify its selection.
- Lines 120–125 contain information more appropriate under "Data Collection Procedure." Consider renaming the subheading to reflect this, or repositioning this section accordingly.

5. **Results and Discussion**

The integration of results and discussion, while common, has in this case limited the depth of discussion. The discussion is brief, lacks adequate citation of supporting or

contrasting literature, and does not sufficiently highlight the implications of the findings. I recommend either separating the results and discussion sections or significantly enhancing the discussion under each theme to provide deeper analysis and context, ultimately supporting stronger recommendations.

6. **Conclusion**

The conclusion currently includes the study's limitations. These would be better placed in a separate section preceding the conclusion.

Additionally:

- The conclusion should focus more explicitly on the study’s aim.
- It is currently lengthy and needs to be more concise and focused.
- Line 583: The statement appears to report quantitative findings, which is inconsistent with the qualitative nature of the study. Consider revising this segment to reflect qualitative insights.
- Lines 588–589 include citations, which are not typically necessary in the conclusion. This section should instead synthesize key findings in relation to the study objectives.

7. **Recommendations**

The implications and recommendations are not clearly derived from the findings. The authors should develop this section further, explicitly linking recommendations to the study’s outcomes. Additionally, areas for future research should be clearly outlined.
