# Supplementary material for: Community health intervention through musical engagement (CHIME) in South Africa: A formative exploration of the feasibility and development of a music-based intervention to support perinatal mental health
Source: PLOS Glob Public Health. 2026 Feb 9;6(2):e0004878. doi: 10.1371/journal.pgph.0004878 (PMC12885302; doi:10.1371/journal.pgph.0004878)
Supplement: Attachment — Submitted filename: Response to reviewers.docx [file pgph.0004878.s007.docx]

**Response to reviewers**

| **Reviewer 1** | | |
| --- | --- | --- |
| # | Comments | Response |
| 1 | The title is generally informative. However, given the qualitative nature of the findings, the study appears to be more exploratory than investigative. I recommend rephrasing the title to reflect this, for example: “...a formative exploration of the…”. | Thank you for this useful suggestion. We have changed the title accordingly. |
| 2 | The CHIME intervention should be clearly defined or operationalized, as it is currently not well explained. | We have added information in the introduction about the intervention in The Gambia (pg 3-4, lines 65-73).  Description of the intervention developed by the Community Health Workers for the South African project is beyond the scope of this paper. We have noted at the end of the study design section that the co-design process and description of the intervention will be subjects of a future paper. (pg 6, lines 38-39) |
| 3 | The description of the study site is minimal. I suggest the authors provide a more comprehensive overview of the study site, including relevant characteristics, to justify its selection. | We have added more detail on the study site as it pertains to the NGO FGDs. (pg 7, lines 158-162) |
| 4 | The integration of results and discussion, has in this case limited the depth of discussion. The discussion is brief, lacks adequate citation of supporting or contrasting literature, and does not sufficiently highlight the implications of the findings. I recommend either separating the results and discussion sections or significantly enhancing the discussion under each theme to provide deeper analysis and context, ultimately supporting stronger recommendations. | Thank you for this feedback. We have expanded the discussion under each theme to provide greater clarity regarding the contributions of the research and to make stronger recommendations based on the findings. See changes starting pg 12. |
| 5 | *On the conclusion*  The conclusion currently includes the study's limitations. These would be better placed in a separate section preceding the conclusion. Additionally:  - The conclusion should focus more explicitly on the study’s aim, not length. - Lines 588–589 include citations, which are not typically necessary in the conclusion. This section should instead synthesize key findings in relation to the study objectives. | Thank you for this, we have placed the limitations at the end of the ‘results and discussion’ section; have retained the original conclusion to remain in the discussion section and have reworded the conclusion to reflect the study aims more explicitly (pg 30-31, lines 736-741). We have removed the citations in this section. |

| **Reviewer 2** | | |
| --- | --- | --- |
| # | Comments | Response |
| 1 | **Line 98-116:** The application of CBPR is to be celebrated here. However, a primary aim of this, is actually transformation and translation of power and decision making within research process. This is not in alignment with what the study actually does - where the external aims and objectives of the project are pre-determined by external stakeholders. Can the authors reflect on this particular tension? How was this mediated by the research team? You make mention of using decolonial feminist frameworks to structure your approach - can you add detail on how this was actually achieved? Is it through analysis? the way questions are defined? Sampling? this detail is needed, and should appear in the methods section of the manuscript. | Clarification has been added regarding the CBPR approach (See pg 6). We have added text in the study design section (and also a few words in the introduction) clarifying the way that the current study:   1. built upon a prior partnership and existing musical practice within the community 2. forms part of a larger CBPR project which included an in-depth co-design process engaging women’s cultural knowledge and expertise as the starting point for intervention development. (While an in-depth discussion of the broader project is outside the scope of this paper, we have added this explanation to clarify how CBPR principles were applied in the research) 3. Detail on how the decolonial feminist approach was implemented is now made more evident in the section on study design and through description in the broader context of the project. |
| 2 | **Line 157**: Your positionally section is very light touch. Intersectionality and decolonial feminist principles would imply a more robust detailing here about researchers - you talk about nationality, but not about any other salient aspects of identity that would contribute to us understanding your positions within the work (i.e. career stages; ethnicity or cultural traditions; personal experiences of motherhood etc). Some of this would be helpful. In line 169-173 you make mention of the co-faciliator role and how it enables senior academics to deviate from formal academia and reflect on their own biases - I think it would be useful to give a specific example of what this looked like in practice. | The detail on positionalities is provided in the supplementary file S3. In addition, we have now added some information on this in the text at the end of this section (pg 10, lines 220 – 226). |
| 3 | **Line 184:** Why were the codes not shared back with the research participants? in CBPR one would anticipate some form of member checking after the analysis, not just for ensuring the circularity of the research process but also to ensure that what was distilled through remained in line with what participants contributed to the process during focus groups. This is a more important form of internal validity, than what the authors refer to in the discussion about potential translation discrepancies - which are important, but not the only important form of validity here. It would also be useful to see a sample coding framework - with detail and examples of how codes and categories shaped each thematic area. | Thank you for this feedback. We have provided clarification in the data analysis section regarding the sharing of codes with participants. (pg 11, lines 243-250)  We have now added a new supplement S4 of a table with the coding framework and example quotes. This is referenced near the beginning of the ‘results and discussion’ section. |
| 4 | **Line 207** - it is not sufficient to tell us that you gain new insights into intergenerational trauma etc. can you elaborate on what these are, or where we can anticipate to find these insights within the remainder of the work that follows? There is too much in this section defining what it is, rather than focusing on what reflexivity resulted in. | Thank you - we have reduced this section and instead added more detail on how reflexivity was enacted as part of the research. (pg 11-12, lines 259-282) |
| 5 | **Line 572**: In the discussion section, It is not customary to start with limitations. The study raises some interesting findings, and it would be more effective to have this come towards the end of the section. | Thank you, as per the other reviewer’s comment we have changed this to come at the end of the ‘results and discussion section’ and have consolidated the findings in the conclusion. |
| 6 | The discussion section could be expanded slightly - are the findings about music and wellbeing in line with, or divergent from what existing evidence says? would the south African approach need to make things different in its delivery from other music interventions used in other contexts? what are the similarities and differences that seem to emerge? The point raised earlier about intergenerational trauma, for example - seems like it is something that should be revisited here - is this pointing us towards needed to more collective/family oriented delivery to acknowledge that trauma is situated across entire family networks - that are often connected to women's caring resources (and thus their mental health outcomes/therapy opportunities?). | Thank you for this feedback. As noted above, we have expanded the discussion section under Themes 1-3 in order to provide more clarity about the contribution of the research and the way in which it aligns with or diverges from existing literature.  We have also included a discussion of music as a cultural determinant of health that may hold particular importance for individuals and communities experiencing the effects of intergenerational trauma, stigma, and isolation (in relation to Theme 2 and Theme 3). |
